# Age- and weight group-specific weight gain patterns in children and adolescents during the 15 years before and during the COVID-19 pandemic

**DOI:** 10.1038/s41366-021-00968-2

**Published:** 2021-09-23

**Authors:** Mandy Vogel, Mandy Geserick, Ruth Gausche, Christoph Beger, Tanja Poulain, Christof Meigen, Antje Körner, Eberhard Keller, Wieland Kiess, Roland Pfäffle

**Affiliations:** 1 Leipzig University Hospital for Children and Adolescents, Leipzig University, Liebigstr. 20a, 04103 Leipzig, Germany; 2 Center for Pediatric Research, Leipzig University, Liebigstr. 20a, 04103 Leipzig, Germany

**Keywords:** Risk factors, Obesity

## Abstract

**Background/Objectives:**

There is a concern that measures aiming to limit a further spread of COVID-19, e.g., school closures and social distancing, cause an aggravation of the childhood obesity epidemic. Therefore, we compared BMI trends during the 15 years before and during the COVID-19 pandemic.

**Subjects/Methods:**

To assess the change in weight dynamics during the first months of COVID-19, we compared the trends of 3-month change in BMI-SDS (ΔBMI-SDS) and the proportions of children showing a high positive (HPC) or high negative (HNC) weight change between 2005 and 2019 and the respective changes from 2019 (pre-pandemic) to 2020 (after the onset of anti-pandemic measures) in more than 150,000 children (9689 during the pandemic period). The period of 3 months corresponds approximately to the first lockdown period in Germany.

**Results:**

During the COVID-19 pandemic, we found a substantial weight gain across all weight and age groups, reflected by an increase in the 3-month change in BMI-SDS (*β* = 0.05, *p* < 0.001), an increase in the proportion of children showing HPC (OR = 1.4, *p* < 0.001), and a decrease in the proportion of children showing HNC (OR = 0.7, *p* < 0.001). Besides, we found the same trends since 2005 on a low but stable level with a yearly increase of ΔBMI-SDS by *β* = 0.001 (*p* < 0.001), the odds of HPC increased by OR_high_pos_ = 1.01 (*p* < 0.001), and the odds of HNC decreased by OR_high_neg_ = 0.99 (*p* < 0.001). These rather small effects accumulated to *β* = 0.02, OR_high_pos_ = 1.14, and OR_high_pos_ = 0.85 over the whole period 2005–2019. Alarmingly, both the long-term and the short-term effects were most pronounced in the obese subgroup.

**Conclusions:**

There are positive dynamics in different measures of weight change, indicating a positive trend in weight gain patterns, especially within the group of children with obesity. These dynamics are likely to be escalated by COVID-19-related measures. Thus, they may lead to a significant further aggravation of the childhood obesity pandemic.

## Introduction

On March 11, 2020, the World Health Organization termed the COVID-19 outbreak a pandemic. Many countries have been affected and took measures to limit infection rates. Those measures included social distancing, home confinement, the closure of shops, sports and cultural facilities, and schools and nurseries. Children, although only mildly affected by the virus itself, experienced profound disruption of their daily lives. Besides adverse psychological effects like increased anxiety and loneliness [1, 2], different scientists worry about an increase in overweight and obesity caused by a decline in physical activity (PA), an increase in sedentary behavior (SB), and a change in dietary behavior [3, 4] associated with increased snacking [5] or higher consumption of ultra-processed food [6]. Indeed, several studies showed a decrease in PA accompanied by an increase in SB in children during the COVID-19-induced confinement [5–8]. Although studies indicated a lockdown-related weight gain in adults [9, 10], to our knowledge, there is no study examining the consequences of the COVID-19-induced behavioral changes on the weight status in a large population-based cohort of children and adolescents covering the whole age range from 1 to 18 years. However, monitoring children’s weight status is of particular interest because childhood obesity is likely to persist into adulthood [11]. With 158,000,000 affected children, childhood obesity is a pandemic on its own [12]. It is related to many comorbidities like hypertension, impaired glucose metabolism, and even cancer [13] or depression [14]; an earlier onset is often related to more severe sequelae [14]. Moreover, in the context of COVID-19, obesity increases the likelihood of severe disease progression, even in children [13, 15].

Based on the weight trend pattern during school time and summer recess, a simulation study predicted an increase in mean BMI-SDS during COVID-19-induced school closures [16]. Other studies showed associations between an increase in overweight/obesity prevalences and economic crises [17, 18] or natural disasters [19]. With more than 500,000,000 children affected by the COVID-19-induced measures [20], even minor effects can cause a tremendous aggravation of the childhood obesity pandemic. Therefore, our study compared the trends of BMI changes and proportions of high positive (HPC)/negative weight changes (HNC) from 2005 to 2019 with the respective changes from 2019 (pre-pandemic) to 2020 (after the onset of anti-pandemic measures) in a large pediatric cohort in Germany.

## Subjects and methods

### Participants and setting

Data were retrieved from the CrescNet patient registry, a network of primary care pediatricians, endocrinological treatment centers, and clinics in Germany. It aims to monitor children’s growth and development for clinical and scientific purposes [21]. All parents gave their informed consent. The data collection is supervised and monitored by the data protection officer of the Medical Faculty, University of Leipzig. CrescNet is registered with ClinicalTrials NCT03072537 as Growth Monitoring Network. Because this study used only retrospective collected data available to the researcher only anonymized, no additional IRB vote was necessary. Height and weight were measured at each visit by trained staff according to standardized procedures [21]. Data on age, sex, height, weight, and diagnoses from any consultation are pseudonymized and transferred to the CrescNet registry. The registry was approved by the Federal Saxonian Data Protection Authority and is registered at ClinicalTrials.gov (NCT03072537). All healthy children aged ≥1 year with a height and weight measurement between September 2019 and February 2020 (t0) and a follow-up measurement between April and July 2020 (t1) were selected from the CrescNet database; the same selection process was done for the years 2005–2019. If children had more than one eligible pair of measurements per year, only the last one was used for analyses. Children with health conditions affecting weight or body composition were not selected. The CrescNet registry contains no information about acute conditions like infectious diseases. The list of excluded diagnoses is included in Supplementary Table 1. There are substantially fewer measurements in 2018–2020. For 2020, there are obvious reasons: (1) there is no baseline measurement between September and December 2020, which leads to fewer included visits/children. (2) There was a drop in visits registered within CrescNet, presumably associated with the COVID-19 and the related measures [22]. Similar effects were observed in different countries, and the suspected reasons are diverse [23, 24]. (3) There is also a delay between measurement and entry to the CrescNet database and the retrospective entry of measurements years after the measurement [21]. The drop from 2017 to 2018 is related to the newly introduced General Data Protection Regulation (legal binding to European law), imposing higher expenses on the participating practices.

### Measures

According to the current German guidelines [25], BMI was calculated and transformed to standard deviation scores (BMI-SDS) using the references by Kromeyer-Hauschild [26]. For each year, the change of BMI-SDS between t0 and t1 was calculated as the standardized difference, i.e., the 3-month change in BMI-SDS (ΔBMI-SDS). Three monthly change rates were chosen because it corresponds to the duration of the first lockdown period in Germany. Classification into the following weight groups was based on BMI-SDS at t0: underweight (UW, <–1.28 BMI-SDS), normal weight (NW, –1.28 ≤ BMI-SDS < 1.28), overweight (OW, 1.28 ≤ BMI-SDS < 1.88), and obese (OB, BMI-SDS ≥ 1.88) also according to the German guidelines [25]. Age at t0 was used to define three age groups: 1–6 years (nursery, preschool), 6–12 (primary school), and 12–18 (secondary school). According to Geserick et al., a change outside ±0.2 BMI-SDS per year was classified as an HPC or an HNC, respectively [11].

### Statistical methods

**Fig. 1:**
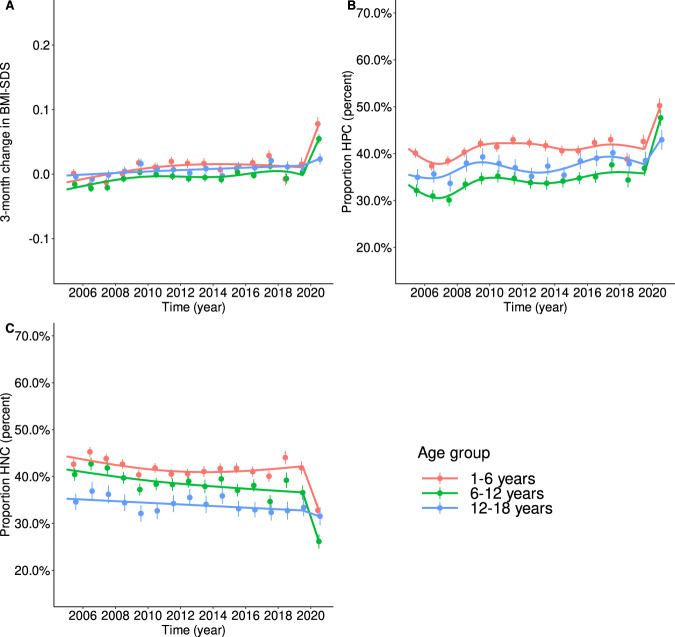
Trends in ΔBMI-SDS, HPC, and HNC between 2005 and 2019 modeled via linear additive mixed models by age group. **A** Trends in ΔBMI-SDS are shown as lines; points indicate yearly means ± 95% confidence levels. Between 2005 and 2019 (pre-pandemic), the trends were mainly stable, showing only a slight increase. From 2019 (prepandemic)to 2020 (after the onset of anti-pandemic measures), we found a substantial increase, less pronounced in the oldest age group. **B** The trends in the proportions of children with HPC show the same pattern as we found for ΔBMI-SDS. The patterns were also similar across the age groups but at higher levels for younger children. **C** Theinverse trends were found for the proportions of children with HNC. There was a small negative trend between 2005 and 2019. Between 2019 (pre-pandemic) and 2020, the proportions of children with HNC decreased substantially. The drop was less pronounced in the oldest age group.

**Fig. 2:**
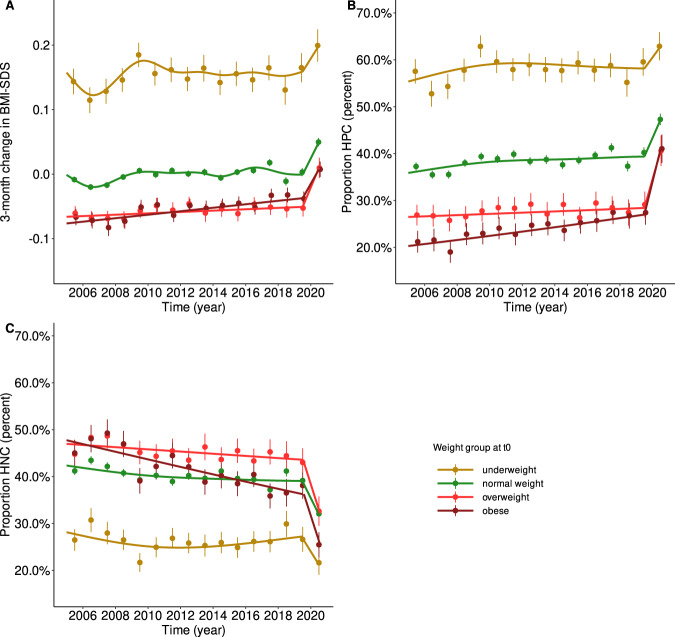
Trends in ΔBMI-SDS, HPC, and HNC between 2005 and 2019 modeled via linear additive mixed models by weight group. **A** Trends in ΔBMI-SDS are shown as lines; points indicate yearly means ± 95% confidence levels. Between 2005 and 2019 (pre-pandemic), the trends were mainly stable, showing only a slight increase. From 2019 (pre-pandemic) to 2020 (after the onset of anti-pandemic measures), we found a substantial increase for all weight groups. **B** The trends in the proportions of children with HPC show the same pattern as we found for ΔBMI-SDS. However, the increase between 2019 (pre-pandemic) and 2020 was substantially stronger for children already affected by overweight and obesity. **C** The inverse trends were found for the proportions of children with HNC. There was a small negative trend between 2005 and 2019 for the normal weight and overweight subgroups. The trend was substantially stronger in children with obesity. Between 2019 (pre-pandemic) and 2020, the proportions of children with HNC decreased substantially. Again, the drop was most pronounced in children already affected by overweight or obesity.

Descriptive statistics were given as mean (standard deviation) for continuous variables and counts (percentage) for categorical variables. To model the time trend of ΔBMI-SDS, generalized additive mixed models (GAMM) were applied with ΔBMI-SDS as the outcome and the numeric equivalent of t1 (centered around 2010 for numerical reasons) as independent variable stratified by weight/age group. Cubic splines were used as smoothing terms. Accordingly, time trends of the proportions of HPC/HNC were estimated applying logistic GAMM. Subsequently, because for all age and weight groups the ΔBMI-SDS and the proportions of HPC/HNC were relatively stable between 2005 and 2019 (Figs. 1 and 2), effects for the respective trends from 2015 to 2019 were estimated using (logistic) linear mixed regression models as was the change between 2019 and 2020. Results are presented as change/odds ratio (OR) per 1 year for the trends between 2005 and 2019, the respective cumulative effect from 2005 to 2019, and as change/OR from 2019 to 2020, including the 95% confidence interval (95% CI). Differences in trends between (a) weight groups, (b) age groups, and (c) boys and girls were examined. To adjust results for multiple measurements per subject, we included the respective random effects in the models. The confidence level was set to *α* = 0.05. *P* values and CIs were adjusted for multiple testing using a method controlling for family-wise error rates as described by Hothorn et al. [27]. All hypothesis tests were two-sided. All statistical analyses were performed using R, v.4.0 [28].

## Results

Data from 274,456 children were retrieved from the CrescNet registry. We excluded 94 measurements from 27 children because of an extreme BMI-SDS (outside the interval [–5, 5]) and 173 measurements from 48 children because they had an extreme change rate (ΔBMI-SDS > 2 for children aged ≥3 or ΔBMI-SDS > 3 for children aged <3 years because smaller children have higher natural variability in BMI-SDS). We considered those extreme values to be likely due to severe organic disorders. Finally, 252,910 measurement pairs from 153,508 children were included in the analyses. The numbers of children included per year, weight, and age group are given in Supplementary Table 2. Basic descriptive statistics by year are given in Table 1. Interestingly, trends in the 3-month change in BMI-SDS differed between age and weight groups but not between sexes (Supplementary Fig. 1). The same was true for percentages of HPC/HNC (Supplementary Figs. 2 and 3). Therefore, sex was not considered during further analyses. In general, the trends differed more between weight groups than between age groups (Figs. 1 and 2). We found no significant interaction between age and weight group.

**Table 1:** Characteristics of the study population stratified by year.

	2005	2006	2007	2008	2009	2010	2011	2012
	*N* = 17,871	*N* = 16,757	*N* = 16,478	*N* = 18,269	*N* = 18,136	*N* = 16,956	*N* = 16,716	*N* = 16,471
*Sex*								
Male	9153 (51%)	8603 (51%)	8407 (51%)	9392 (51%)	9609 (53%)	8935 (53%)	8794 (53%)	8744 (53%)
Female	8718 (49%)	8154 (49%)	8071 (49%)	8877 (49%)	8527 (47%)	8021 (47%)	7922 (47%)	7727 (47%)
*Age t0 (years)*	6.89 (4.41)	6.70 (4.31)	6.77 (4.41)	6.79 (4.31)	6.72 (4.35)	6.87 (4.37)	6.91 (4.39)	6.86 (4.43)
*ΔAge (years)*	0.50 (0.16)	0.50 (0.16)	0.50 (0.16)	0.48 (0.16)	0.48 (0.17)	0.48 (0.17)	0.48 (0.16)	0.49 (0.16)
*Weight group (t0)*								
Underweight	1465 (8%)	1321 (8%)	1423(9%)	1653 (9%)	1715 (9%)	1656 (10%)	1635 (10%)	1622 (10%)
Normal weight	13,528 (76%)	12,819 (77%)	12,491 (76%)	13,743 (75%)	13,534 (75%)	12,484 (74%)	12,321 (74%)	11,974 (73%)
Overweight	1643 (9%)	1440 (9%)	1397 (8%)	1585 (9%)	1595 (9%)	1509 (9%)	1456 (9%)	1492 (9%)
Obese	1235 (7%)	1177 (7%)	1167 (7%)	1288 (7%)	1292 (7%)	1307 (8%)	1304 (8%)	1383 (8%)
*BMI-SDS t0*	0.19 (1.11)	0.18 (1.09)	0.18 (1.11)	0.17 (1.12)	0.15 (1.14)	0.17 (1.15)	0.16 (1.16)	0.19 (1.16)
*BMI-SDS t1*	0.17 (1.10)	0.15 (1.10)	0.15 (1.11)	0.15 (1.11)	0.16 (1.12)	0.17 (1.14)	0.17 (1.14)	0.19 (1.16)
*ΔBMI-SDS/3 months*	0.00 (0.27)	–0.02 (0.27)	–0.01 (0.27)	0.00 (0.28)	0.01 (0.29)	0.01 (0.29)	0.01 (0.29)	0.01 (0.28)
	2013	2014	2015	2016	2017	2018	2019	2020
	*N* = 13,954	*N* = 15,667	*N* = 17,148	*N* = 17,207	*N* = 15,010	*N* = 10,703	*N* = 11,272	*N* = 9689
*Sex*								
Male	7463 (54%)	8246 (53%)	9101 (53%)	9116 (53%)	7822 (52%)	5279 (49%)	5921 (53%)	5248 (54%)
Female	6491 (47%)	7421 (47%)	8047 (47%)	8091 (47%)	7188 (48%)	5424 (51%)	5351 (47%)	4441 (46%)
*Age t0 (years)*	7.14 (4.51)	7.24 (4.55)	7.12 (4.52)	7.18 (4.56)	7.39 (4.55)	7.73 (4.67)	7.75 (4.66)	7.83 (4.70)
*ΔAge (years)*	0.48 (0.17)	0.48 (0.17)	0.48 (0.16)	0.47 (0.16)	0.46 (0.16)	0.46 (0.16)	0.46 (0.17)	0.50 (0.16)
*Weight group (t0)*								
Underweight	1371 (10%)	1473 (9%)	1568 (9%)	1613 (9%)	1500 (10%)	1080 (10%)	1120 (10%)	1040 (11%)
Normal weight	10,053 (72%)	11,410 (73%)	12,728 (74%)	12,782 (74%)	10,817 (72%)	7526 (70%)	7882 (70%)	6688 (69%)
Overweight	1235 (9%)	1430 (9%)	1496 (9%)	1442 (8%)	1394 (9%)	1024 (10%)	1081 (10%)	901 (9%)
Obese	1295 (9%)	1354 (9%)	1356 (8%)	1370 (8%)	1299 (9%)	1073 (10.0%)	1189 (10%)	1060 (11%)
*BMI-SDS t0*	0.19 (1.19)	0.19 (1.18)	0.18 (1.15)	0.17 (1.16)	0.19 (1.19)	0.23 (1.24)	0.23 (1.24)	0.24 (1.26)
*BMI-SDS t1*	0.20 (1.18)	0.18 (1.17)	0.19 (1.14)	0.18 (1.15)	0.21 (1.18)	0.22 (1.23)	0.25 (1.22)	0.33 (1.26)
*ΔBMI-SDS/3 months*	0.01 (0.28)	0.00 (0.28)	0.01 (0.28)	0.01 (0.28)	0.02 (0.29)	0.00 (0.28)	0.01 (0.29)	0.06 (0.28)

### Trends in the 3-month change of BMI-SDS

Overall, there was a yearly increase of *β*_1y_ = 0.0014 (95% CI 0.0011–0.0017; *p* < 0.001) in the 3-month change in BMI-SDS between 2005 and 2019, i.e., the 3-month change in BMI-SDS increased between 2005 and 2019 by 0.02 SDS from –0.006 in 2005 to 0.013 in 2019. The increase was similar across the age groups but differed significantly across weight groups. Whereas the groups UW, NW, and OW showed marginally significant effects around *β*_1y_ = 0.001, a substantially higher effect was present in the OB group (*β*_1y_ = 0.003, 95% CI 0.001–0.004; *p* < 0.001). From 2019 to 2020, we found an increase of *β* = 0.048 in the 3-month change in BMI-SDS (95% CI 0.039–0.056; *p* < 0.001)—more than 30 times higher than the rate before 2020. Therefore, in 2020, the resulting 3-month change in BMI-SDS was 0.06. The increase was higher in younger children (1–6 years: *β* = 0.062; 95% CI 0.047–0.078; *p* < 0.001; 6–12 years: *β* = 0.051; 95% CI 0.034–0.069; *p* < 0.001). In adolescents, the effect did not reach statistical significance. The trends are visualized in Figs. 1A and 2A.

### Trends in high positive (HPC) and high negative (HPC) change in BMI-SDS

Notably, from 2005 to 2019, there was a positive trend (OR_1y_ = 1.01 per year; 95% CI 1.009–1.011; *p* < 0.001) in the proportion of children with an HPC and a negative one (OR_1y_ = 0.99 per year; 95% CI 0.987–0.992; *p* < 0.001) in the proportion of children with an HNC. These effects accumulated to OR_15y_ = 1.14 (95% CI 1.10–1.18) and OR_15y_ = 0.85 (95% CI 0.82–0.88), whereas we found no difference in the trend between age groups for HPC, the trend for HNC was stronger in 6- to 12-year-old children (OR_15y_ = 0.81; 95% CI 0.76–0.87; *p* < 0.001). Considering the weight status, both effects, HPC and HNC, were substantially stronger in the OB group (HPC: OR_15y_ = 1.47; 95% CI 1.25–1.73; *p* < 0.001; HNC: OR_15y_ = 0.61; 95% CI 0.53–0.71; *p* < 0.001), which is consistent with the higher increase in the 3-month change in BMI-SDS in this group. For HPC, the cumulative effects in UW, NW, and OW varied between ORs_15y_ of 1.07 and 1.15, reaching the level for significance only for NW. For HNC, we found also smaller but similar effects for NW (OR_15y_ = 0.87, 95% CI 0.83–0.91; *p* < 0.001) and OW (OR_15y_ = 0.87; 95% CI 0.76–0.99; *p* = 0.033). When looking at the respective percentages of children with HPC and HNC, we found that the percentages of children with HNC varied between 45% and 50% in the OW and OB weight group until 2008 and decreased to around 40% in 2019. On the other hand, there was an increase of children with overweight or obesity showing HPC: from about 26% (OW)/22% (OB) until 2008 to 29% (OW)/28% (OB) in 2019 (pre-pandemic).

From 2019 to 2020, the increase in the proportion of children with HPC corresponds to an OR of 1.38 (95% CI 1.30–1.47; *p* < 0.001). As for the 3-month change in BMI-SDS, the effect is more than 30 times as high as for the years before. A similar effect was observed for the proportion of children showing HNC (OR = 0.70; 95% CI 0.66–0.75; *p* < 0.001). Both effects were stronger for higher weight status. Hence, the highest effects were found in the OB group. Here, the OR for HPC was OR_high_pos_ = 1.85 (95% CI 1.45–2.35; *p* < 0.001), and for HNC, it was OR_high_neg_ = 0.55 (95% CI 0.43–0.71; *p* < 0.001). For OW, the respective effects were OR_high_pos_ = 1.67 (95% CI 1.30–2.16; *p* < 0.001) and OR_high_neg_ = 0.64 (95% CI 0.50–0.82; *p* < 0.001), and for NW, OR_high_pos_ = 1.34; 95% CI 1.22–1.46; *p* < 0.001) and OR_high_neg_ = 0.73 (95% CI 0.67–0.80; *p* < 0.001). For UW, only the proportion of children showing HNC changed significantly, with an OR_high_neg_ = 0.76 (95% CI 0.58–0.99; *p* = 0.044). Considering age, the effects were more pronounced in children and younger adolescents (1–6 years: OR_high_pos_ = 1.36; 95% CI 1.22–1.52; *p* < 0.001 and OR_high_neg_ = 0.68; 95% CI 0.60–0.76; *p* < 0.001; 6–12 years: OR_high_pos_ = 1.56; 95% CI 1.38–1.77; *p* < 0.001 and OR_high_neg_ = 0.61; 95% CI 0.54–0.70; *p* < 0.001). In adolescents, only the effect for an HPC reached statistical significance (OR_high_pos_ = 1.20; 95% CI 1.03–1.40; *p* = 0.010).

Subsequently, during the first few months of the pandemic, the proportion of children with HNC shrunk by more than 10% and reached 33% and 26% in the OW and OB weight group, respectively. At the same time, the proportions of children with HPC rose by more than 10–41% (OW and OB), which is consistent with the higher pandemic change in BMI-SDS.

All effects (overall as well as stratified by age and weight group) are summarized in Table 2 and visualized in Figs. 1B, C and 2B, C. For the interest of the reader, the prevalences of UW, NW, OW, and OB are given stratified by age and weight group in Supplementary Table 3.

**Table 2:** Overview of the effects for trends between 2005 and 2019 and between 2019 and 2020.

	ΔBMI-SDS			Proportion high positive			Proportion high negative		
	*β* (95% CI)		*p* value	*β* (95% CI)		*p* value	*β* (95% CI)		*p* value
	Per 1 year	Cumulative effect 2005–2019		Per 1 year	Cumulative effect 2005–2019		Per 1 year	Cumulative effect 2005–2019	
*Effects 2005–2019*									
Overall	0.001 [0.001, 0.002]	0.021 [0.017, 0.026]	*p* < 0.001	1.009 [1.007, 1.011]	1.142 [1.104, 1.181]	*p* < 0.001	0.989 [0.987, 0.992]	0.851 [0.823, 0.880]	*p* < 0.001
Per age group									
1–6 years	0.002 [0.001, 0.002]	0.025 [0.017, 0.032]	*p* < 0.001	1.008 [1.004, 1.012]	1.126 [1.065, 1.191]	*p* < 0.001	0.994 [0.990, 0.997]	0.907 [0.859, 0.959]	*p* < 0.001
6–12 years	0.001 [0.001, 0.002]	0.021 [0.012, 0.031]	*p* < 0.001	1.014 [1.010, 1.019]	1.239 [1.153, 1.332]	*p* < 0.001	0.986 [0.982, 0.991]	0.813 [0.758, 0.872]	*p* < 0.001
12–18 years	0.001 [0.000, 0.002]	0.015 [0.003, 0.028]	*p* = 0.007	1.010 [1.004, 1.016]	1.163 [1.059, 1.276]	*p* < 0.001	0.992 [0.986, 0.999]	0.891 [0.811, 0.979]	*p* = 0.008
Per weight group									
Underweight	0.001 [–0.000, 0.002]	0.015 [–0.003, 0.033]	*p* = 0.166	1.005 [0.996, 1.014]	1.074 [0.939, 1.228]	*p* = 0.651	0.999 [0.989, 1.008]	0.978 [0.843, 1.135]	*p* = 0.999
Normal weight	0.001 [0.001, 0.002]	0.020 [0.013, 0.026]	*p* < 0.001	1.009 [1.006, 1.012]	1.147 [1.093, 1.203]	*p* < 0.001	0.991 [0.988, 0.994]	0.874 [0.834, 0.916]	*p* < 0.001
Overweight	0.001 [–0.000, 0.002]	0.016 [–0.002, 0.034]	*p* = 0.117	1.007 [0.997, 1.017]	1.105 [0.953, 1.281]	*p* = 0.374	0.991 [0.982, 1.000]	0.870 [0.762, 0.993]	*p* = 0.033
Obese	0.003 [0.001, 0.004]	0.041 [0.021, 0.060]	*p* < 0.001	1.026 [1.015, 1.037]	1.470 [1.248, 1.733]	*p* < 0.001	0.968 [0.959, 0.977]	0.612 [0.531, 0.705]	*p* < 0.001
*Effects 2019–2020*									
Overall	0.048 [0.039, 0.056]		*p* < 0.001	1.384 [1.301, 1.472]		*p* < 0.001	0.702 [0.658, 0.750]		*p* < 0.001
Per age group									
1–6 years	0.062 [0.047, 0.078]		*p* < 0.001	1.364 [1.221, 1.522]		*p* < 0.001	0.677 [0.604, 0.759]		*p* < 0.001
6–12 years	0.051 [0.034, 0.069]		*p* < 0.001	1.562 [1.377, 1.773]		*p* < 0.001	0.613 [0.535, 0.703]		*p* < 0.001
12–18 years	0.016 [–0.005, 0.037]		*p* = 0.235	1.203 [1.032, 1.402]		*p* = 0.010	0.918 [0.782, 1.078]		*p* = 0.582
Per weight group									
Underweight	0.035 [0.004, 0.067]		*p* = 0.019	1.153 [0.910, 1.461]		*p* = 0.491	0.760 [0.580, 0.995]		*p* = 0.044
Normal weight	0.046 [0.034, 0.059]		*p* < 0.001	1.337 [1.222, 1.463]		*p* < 0.001	0.732 [0.667, 0.804]		*p* < 0.001
Overweight	0.062 [0.029, 0.095]		*p* < 0.001	1.670 [1.294, 2.155]		*p* < 0.001	0.640 [0.498, 0.822]		*p* < 0.001
Obese	0.044 [0.014, 0.075]		*p* < 0.001	1.850 [1.453, 2.354]		*p* < 0.001	0.554 [0.433, 0.709]		*p* < 0.001

## Discussion

This registry-based study found a small but stable positive trend in the 3-month change in BMI-SDS between 2005 and 2020, with an exceptional aggravation during the last year—the latter most likely attributable to COVID-19-induced measures. Our finding of an accelerated change in BMI-SDS between 2005 and 2019 is not contrary to studies reporting stabilizing or even downward trends of overweight and obesity prevalence [29, 30]. Rather, we found the most pronounced dynamics within the already affected population. In general, the trend was positive for all age and weight groups and strongest in children already affected by overweight or obesity. Even if some subgroup effects did not reach statistical significance, this stable consistency is alarming: especially in children with obesity, we would expect some negative effects because of the statistical regression to the mean phenomenon, i.e., if we observe some extreme values (like BMI-SDS values in the range of obesity), we would expect a future value being less extreme and, therefore, nearer to the mean. The aggravation of childhood obesity becomes even more evident when we look at the proportions of children with HPC or HNC. The percentage of children with HNC fell from approximately 45–50% (2005–2008) in the OW and OB weight group to around 40% in 2019, whereas the percentage of children with HPC rose from about 26% (OW)/22% (OB) to 29% (OW)/28% (OB) in 2019 (pre-pandemic).

The effects from 2019 to 2020 are even more alarming. Despite the short time interval of a few months, they surmounted the cumulative effects of the 15 years before: the percentage of children with HPC/HNC rose/dropped by more than 10% for children with overweight or obesity. Even though adolescents seem to be less severely affected, there is no reason for giving the all-clear: the obesity prevalence in this age group increased from 2005 to 2020 (pre-pandemic) from 10% to 19% (Supplementary Table 3). Older adolescents are the age group with the highest obesity prevalence. Here, it becomes evident that even a small increase can have large effects over time. The same effects were found in normal weight children but less pronounced. The already most affected population seems to be the most vulnerable, whether in terms of small long-term or short-term effects.

Our findings are in line with several studies on COVID-19-related weight changes in adults [9, 10] and children [31–34] and studies on weight gain in children associated with the economic crisis in 2007–2008 [17, 18, 35] or the great Fukushima earthquake 2011 [19, 36]. Zheng et al. showed that the initial increase of BMI and obesity prevalence persisted or even aggravated at least two years after the earthquake. The effect was more pronounced if children also experienced a personal disaster like a stay in an evacuation center or the death of a family member [36]. Indeed, a Korean study in 226 pediatric patients of a growth clinic found a mean BMI-SDS change of approximately +0.22 SDS/year during the lockdown accompanied by higher LDL, triglyceride, uric acid, and total cholesterol values [37]. The change rate is comparable to our 3-month change in BMI-SDS of +0.06 SDS that corresponds to a yearly change of +0.24 SDS. Weaver et al. found an even greater BMI-SDS change of +0.34 SDS/year in 182 US-American elementary school children [33]. Wen et al. examined almost 20,000 Chinese preschoolers aged 3–5.5 years. They also found higher BMI change rates during the first pandemic lockdown period than during the two years before [31]. Brooks et al. analyzed BMI data from more than 45,000 6- to 17-year-old children and adolescents between September and December 2020 and compared the changes rates to pre-pandemic changes rates (2017 to March 2020). In line with our results, they found an excessive acceleration of BMI-SDS change in the younger children, with rates two times as high as before. Moreover, they also found children with overweight and obesity more severely affected than their normal weight peers [34]. In addition, several other studies reported a remarkable weight gain in children associated with COVID-related lockdown periods (Korea, *n* = 169, pediatric patients [38]; US, *n* = 29, elementary school children [39]; China, *n* = 445, children aged 7–12 years [40]; China, *n* = 10,082, students of high schools, colleges, and graduate schools aged >16 years [41]; Italy, *n* = 51, adolescents with obesity aged 10–18 [32]; Greece *n* = 397, children and adolescents aged 2–18 years [42]).

Although we have no information on the health-related behavior regarding our study population, a decrease in PA and an unfavorable change in dietary behavior seem to be the most likely explanation for the sudden and high change in weight trends. Germany was mildly to moderately affected during the first COVID-19 wave. However, in late March 2020, non-essential educational, cultural, and administrative facilities were closed. Only essential shops stayed open. Playgrounds and sports facilities were closed and cordoned off. Schools and nurseries stayed entirely closed until mid-May. Education was only possible through online platforms. From mid-May, a partial re-opening was allowed with restrictions (hygiene rules, distancing, wearing masks, strict separation of groups, etc., had to be secured). Most schools implemented a hybrid model of online and on-site lessons. The proportion of on-site classes was often less than 50%. Besides, compulsory education was suspended until the end of the school year, i.e., parents could decide whether or not a child had to go to school. Nurseries were also re-opened in mid-May, but opening hours were often limited due to staff shortages (employees belonged to a risk group, childcare obligations, COVID-19-related quarantine) [43]. Children and adolescents were living through a time of rapid changes in their daily routines. For many, the days became less structured, which is known to increases the risk of obesogenic behaviors [44, 45]. And indeed, several studies showed an accelerated weight gain during the summer months compared to the school year and time in nurseries [45, 46], with higher effects in overweight than in normal weight children [47]. The mechanisms are not fully understood [45]. A COVID-19-related decrease in PA has already been shown [5–8, 48–50], often related to an increase of SB [5, 7, 8, 49, 50]. There are mixed results concerning dietary behavior [5, 50, 51]. Several studies reported higher consumption of fruits or vegetables [5, 42, 51–53], one lower [50]. There was also an increase in the consumption of unhealthy foods and sweets [5, 51]. The latter might be tightly connected to increased snacking [9, 52, 53] during the increased screen time [4, 54] or due to boredom [51]. This is in line with studies in adults, reporting an increase in food consumption because of increased snacking, due to boredom, and the increased availability of food during the COVID-19-induced confinement [9, 55]. Other reasons for increased snacking in children might be changes in parental feeding behavior. Jansen et al. and Philippe et al. showed increased emotional and instrumental feeding practices in parents of 2- to 12-year-old children during COVID-19 [52, 56].

### Limitations

Participation in the CrescNet registry is voluntary. We have no information of non-participants, neither of the number of non-participants nor the reasons for non-participation. Furthermore, we have no information on behavioral patterns like PA, sedentary time, eating behavior, or food consumption. Therefore, we cannot examine the associations between changes in weight gain pattern and possible reasons or other possible confounders, except age, sex, and initial weight group. We also cannot guarantee that the composition of the population did not change due to COVID-19 in regard to the unmeasured potential confounders (e.g., socioeconomic status, PA, nutritional behavior).

## Conclusion

We found a small but stable positive trend in the 3-month change in BMI-SDS and in the proportion of children with a high positive weight change between 2005 and 2020. These trends were accompanied by a decrease in the proportion of children with a high negative weight change, both adding to the still-growing problem of childhood obesity. During the COVID-19 pandemic, these effects have increased by more than 30 times within the relatively short period. We hypothesize that these effects are caused by the COVID-19-induced changes in health-related behavior and may lead to a significant further aggravation of the childhood obesity pandemic.

## Supplementary information

Supplementary Material
