# Supplementary material for: Age- and weight group-specific weight gain patterns in children and adolescents during the 15 years before and during the COVID-19 pandemic
Source: Int J Obes (Lond). 2021 Sep 23;46(1):144–52. doi: 10.1038/s41366-021-00968-2 (PMC8458556; doi:10.1038/s41366-021-00968-2)

**Online-Only Supplements**

### Content

Supplentary Tables

Supplementary Table 1:

ICD10 codes and descriptions of diagnoses excluded from the database query.

Supplementary Table 2:

The numbers of included children stratified by age and weight group

Supplementary Table 3:

Prevalences of underweight, normal weight, overweight, and obesity

stratified by age

Supplementary Figures

Supplementary Figure 1:

ΔBMI-SDS trends within the age and weight groups stratified by sex

Supplementary Figure 2:

Trends of the proportion of children gaining weight stratified by sex

Supplementary Figure 3:

Trends of the proportion of children losing weight stratified by sex

### Supplemantary Tables

Supplementary Table 1: ICD10 codes and descriptions of diagnoses excluded from the database query.

| ICD10 code starts with | description |
| --- | --- |
| C | malignant neoplasm |
| D33 | benign neoplasm of meninges |
| D35 | benign neoplasm endocrine glands |
| D82.2 | Immunodeficiency with short-limbed stature |
| E03 | hypothyroidism |
| E20/E21 | hypoparathyroidism andother disorders of parathyroid gland |
| E22.0 | acromegaly and pituitary gigantism |
| E23 | hypofunction and other disorders of pituitary gland |
| E24 | cushing syndrome |
| E25 | adrenogenital disorders |
| E26 | hyperaldosteronism |
| E27 | other disorders of adrenal gland |
| E30.1 | disorders of puberty |
| E7, E8, E90 | metabolic disorders |
| G80-G83 | Cerebral palsy and other paralytic syndromes |
| K50-K52 | noninfective enteritis and colitis |
| K90 | intestinal malabsorption |
| N25 | disorders resulting from impaired renal tubular function |
| Q77/Q78 | osteochondrodysplasia |
| Q86 | congenital malformation syndromes due to known exogenous causes |
| Q87 | other specified congenital malformation syndromes affecting multiple systems |
| Q89 | other specified congenital |
| Q9 | other chromosomal abnormalities |

Supplementary Table 2: The numbers of included children per age and weight group for each of the considered years.

|  | age group | | | | | | | | | | | | | | |
| --- | --- | --- | --- | --- | --- | --- | --- | --- | --- | --- | --- | --- | --- | --- | --- |
|  | 1-6 years | | | | | 6-12 years | | | | | 12-18 years | | | | |
| year | under- weight | normal weight | over- weight | obese | **sum** | under- weight | normal weight | over- weight | obese | **sum** | under- weight | normal weight | over- weight | obese | **sum** |
| 2005 | 763 | 7592 | 698 | 411 | **9464** | 410 | 3786 | 606 | 505 | **5307** | 292 | 2150 | 339 | 319 | **3100** |
| 2006 | 703 | 7285 | 648 | 353 | **8989** | 392 | 3760 | 520 | 541 | **5213** | 226 | 1774 | 272 | 283 | **2555** |
| 2007 | 732 | 7118 | 612 | 372 | **8834** | 432 | 3530 | 517 | 492 | **4971** | 259 | 1843 | 268 | 303 | **2673** |
| 2008 | 847 | 7602 | 677 | 407 | **9533** | 518 | 4219 | 624 | 555 | **5916** | 288 | 1922 | 284 | 326 | **2820** |
| 2009 | 933 | 7730 | 674 | 388 | **9725** | 502 | 3948 | 609 | 545 | **5604** | 280 | 1856 | 312 | 359 | **2807** |
| 2010 | 887 | 6904 | 600 | 350 | **8741** | 489 | 3831 | 579 | 595 | **5494** | 280 | 1749 | 330 | 362 | **2721** |
| 2011 | 826 | 6778 | 600 | 325 | **8529** | 549 | 3724 | 553 | 584 | **5410** | 260 | 1819 | 303 | 395 | **2777** |
| 2012 | 804 | 6791 | 579 | 338 | **8512** | 531 | 3478 | 569 | 610 | **5188** | 287 | 1705 | 344 | 435 | **2771** |
| 2013 | 698 | 5411 | 457 | 305 | **6871** | 415 | 3034 | 475 | 529 | **4453** | 258 | 1608 | 303 | 461 | **2630** |
| 2014 | 736 | 6026 | 508 | 334 | **7604** | 442 | 3482 | 564 | 512 | **5000** | 295 | 1902 | 358 | 508 | **3063** |
| 2015 | 746 | 6797 | 585 | 363 | **8491** | 477 | 3899 | 557 | 548 | **5481** | 345 | 2032 | 354 | 445 | **3176** |
| 2016 | 752 | 6730 | 579 | 350 | **8411** | 529 | 3921 | 533 | 554 | **5537** | 332 | 2131 | 330 | 466 | **3259** |
| 2017 | 667 | 5500 | 501 | 302 | **6970** | 510 | 3444 | 555 | 537 | **5046** | 323 | 1873 | 338 | 460 | **2994** |
| 2018 | 470 | 3644 | 346 | 250 | **4710** | 370 | 2390 | 421 | 402 | **3583** | 240 | 1492 | 257 | 421 | **2410** |
| 2019 | 499 | 3804 | 360 | 253 | **4916** | 351 | 2554 | 425 | 492 | **3822** | 270 | 1524 | 296 | 444 | **2534** |
| 2020 | 482 | 3201 | 274 | 213 | **4170** | 321 | 2136 | 376 | 430 | **3263** | 237 | 1351 | 251 | 417 | **2256** |

Supplementary Table 3: Prevalences of underweight, normal weight, overweight, and obesity stratified by age and weight group at t0 and t1 for each of the considered years.

|  | underweight | | | | | | normal weight | | | | | |
| --- | --- | --- | --- | --- | --- | --- | --- | --- | --- | --- | --- | --- |
|  | 1-6-years old | | 6-12-years old | | 12-18-years old | | 1-6-years old | | 6-12-years old | | 12-18-years old | |
| year | % at t0 | % at t1 | % at t0 | % at t1 | % at t0 | % at t1 | % at t0 | % at t1 | % at t0 | % at t1 | % at t0 | % at t1 |
| 2005 | 8.1 | 7.7 | 7.7 | 8.3 | 9.4 | 9.3 | 80.2 | 81.2 | 71.3 | 71.7 | 69.4 | 69.6 |
| 2006 | 7.8 | 8.6 | 7.5 | 9.0 | 8.8 | 9.4 | 81.0 | 80.9 | 72.1 | 71.2 | 69.4 | 69.6 |
| 2007 | 8.3 | 8.6 | 8.7 | 9.3 | 9.7 | 9.2 | 80.6 | 81.0 | 71.0 | 70.9 | 68.9 | 69.5 |
| 2008 | 8.9 | 8.2 | 8.8 | 9.2 | 10.2 | 9.2 | 79.7 | 81.2 | 71.3 | 71.1 | 68.2 | 69.2 |
| 2009 | 9.6 | 9.0 | 9.0 | 8.8 | 10.0 | 9.0 | 79.5 | 80.4 | 70.4 | 70.7 | 66.1 | 67.3 |
| 2010 | 10.1 | 9.1 | 8.9 | 9.0 | 10.3 | 9.8 | 79.0 | 80.4 | 69.7 | 69.5 | 64.3 | 64.9 |
| 2011 | 9.7 | 8.6 | 10.1 | 10.0 | 9.4 | 9.4 | 79.5 | 80.8 | 68.8 | 68.6 | 65.5 | 65.6 |
| 2012 | 9.4 | 9.1 | 10.2 | 10.1 | 10.4 | 9.7 | 79.8 | 79.9 | 67.0 | 67.5 | 61.5 | 62.4 |
| 2013 | 10.2 | 8.7 | 9.3 | 9.5 | 9.8 | 9.5 | 78.8 | 80.3 | 68.1 | 68.0 | 61.1 | 61.5 |
| 2014 | 9.7 | 9.1 | 8.8 | 8.8 | 9.6 | 9.5 | 79.2 | 80.1 | 69.6 | 70.2 | 62.1 | 62.3 |
| 2015 | 8.8 | 8.2 | 8.7 | 8.6 | 10.9 | 10.5 | 80.0 | 80.9 | 71.1 | 71.3 | 64.0 | 64.4 |
| 2016 | 8.9 | 8.0 | 9.6 | 10.0 | 10.2 | 10.0 | 80.0 | 80.6 | 70.8 | 70.2 | 65.4 | 64.7 |
| 2017 | 9.6 | 8.1 | 10.1 | 9.5 | 10.8 | 10.7 | 78.9 | 80.0 | 68.3 | 69.0 | 62.6 | 62.4 |
| 2018 | 10.0 | 9.7 | 10.3 | 10.5 | 10.0 | 10.1 | 77.4 | 78.1 | 66.7 | 66.5 | 61.9 | 61.5 |
| 2019 | 10.2 | 9.6 | 9.2 | 8.9 | 10.7 | 10.1 | 77.4 | 78.6 | 66.8 | 66.8 | 60.1 | 61.1 |
| 2020 | 11.6 | 9.1 | 9.8 | 8.9 | 10.5 | 9.6 | 76.8 | 76.8 | 65.5 | 63.5 | 59.9 | 60.7 |
|  | overweight | | | | | | obese | | | | | |
|  | 1-6-years old | | 6-12-years old | | 12-18-years old | | 1-6-years old | | 6-12-years old | | 12-18-years old | |
|  | % at t0 | % at t1 | % at t0 | % at t1 | % at t0 | % at t1 | % at t0 | % at t1 | % at t0 | % at t1 | % at t0 | % at t1 |
| 2005 | 7.4 | 6.8 | 11.4 | 10.9 | 10.9 | 10.5 | 4.3 | 4.3 | 9.5 | 9.1 | 10.3 | 10.6 |
| 2006 | 7.2 | 6.8 | 10.0 | 10.5 | 10.6 | 10.1 | 3.9 | 3.7 | 10.4 | 9.3 | 11.1 | 11.0 |
| 2007 | 6.9 | 6.6 | 10.4 | 10.8 | 10.0 | 10.6 | 4.2 | 3.7 | 9.9 | 9.0 | 11.3 | 10.7 |
| 2008 | 7.1 | 6.6 | 10.5 | 10.8 | 10.1 | 10.0 | 4.3 | 4.1 | 9.4 | 8.9 | 11.6 | 11.6 |
| 2009 | 6.9 | 6.6 | 10.9 | 10.7 | 11.1 | 10.9 | 4.0 | 4.0 | 9.7 | 9.7 | 12.8 | 12.8 |
| 2010 | 6.9 | 6.1 | 10.5 | 11.4 | 12.1 | 11.8 | 4.0 | 4.3 | 10.8 | 10.1 | 13.3 | 13.5 |
| 2011 | 7.0 | 6.9 | 10.2 | 11.3 | 10.9 | 11.1 | 3.8 | 3.8 | 10.8 | 10.1 | 14.2 | 13.9 |
| 2012 | 6.8 | 6.8 | 11.0 | 11.1 | 12.4 | 13.1 | 4.0 | 4.1 | 11.8 | 11.3 | 15.7 | 14.9 |
| 2013 | 6.7 | 6.6 | 10.7 | 11.1 | 11.5 | 11.6 | 4.4 | 4.4 | 11.9 | 11.4 | 17.5 | 17.4 |
| 2014 | 6.7 | 6.4 | 11.3 | 10.8 | 11.7 | 11.9 | 4.4 | 4.4 | 10.2 | 10.1 | 16.6 | 16.3 |
| 2015 | 6.9 | 6.7 | 10.2 | 10.3 | 11.1 | 11.0 | 4.3 | 4.3 | 10.0 | 9.8 | 14.0 | 14.1 |
| 2016 | 6.9 | 7.4 | 9.6 | 10.1 | 10.1 | 10.9 | 4.2 | 4.0 | 10.0 | 9.6 | 14.3 | 14.4 |
| 2017 | 7.2 | 7.4 | 11.0 | 10.6 | 11.3 | 11.7 | 4.3 | 4.4 | 10.6 | 10.8 | 15.4 | 15.3 |
| 2018 | 7.3 | 7.1 | 11.7 | 11.6 | 10.7 | 10.2 | 5.3 | 5.1 | 11.2 | 11.3 | 17.5 | 18.2 |
| 2019 | 7.3 | 6.6 | 11.1 | 11.7 | 11.8 | 11.0 | 5.1 | 5.1 | 12.9 | 12.5 | 17.5 | 17.6 |
| 2020 | 6.6 | 7.6 | 11.5 | 13.1 | 11.1 | 10.8 | 5.1 | 6.4 | 13.2 | 14.6 | 18.5 | 18.9 |

## Supplementary Figures

eFigure 1: ΔBMI-SDS trends within the age and weight groups stratified by sex: Interestingly, we found no difference in trends between boys and girls.


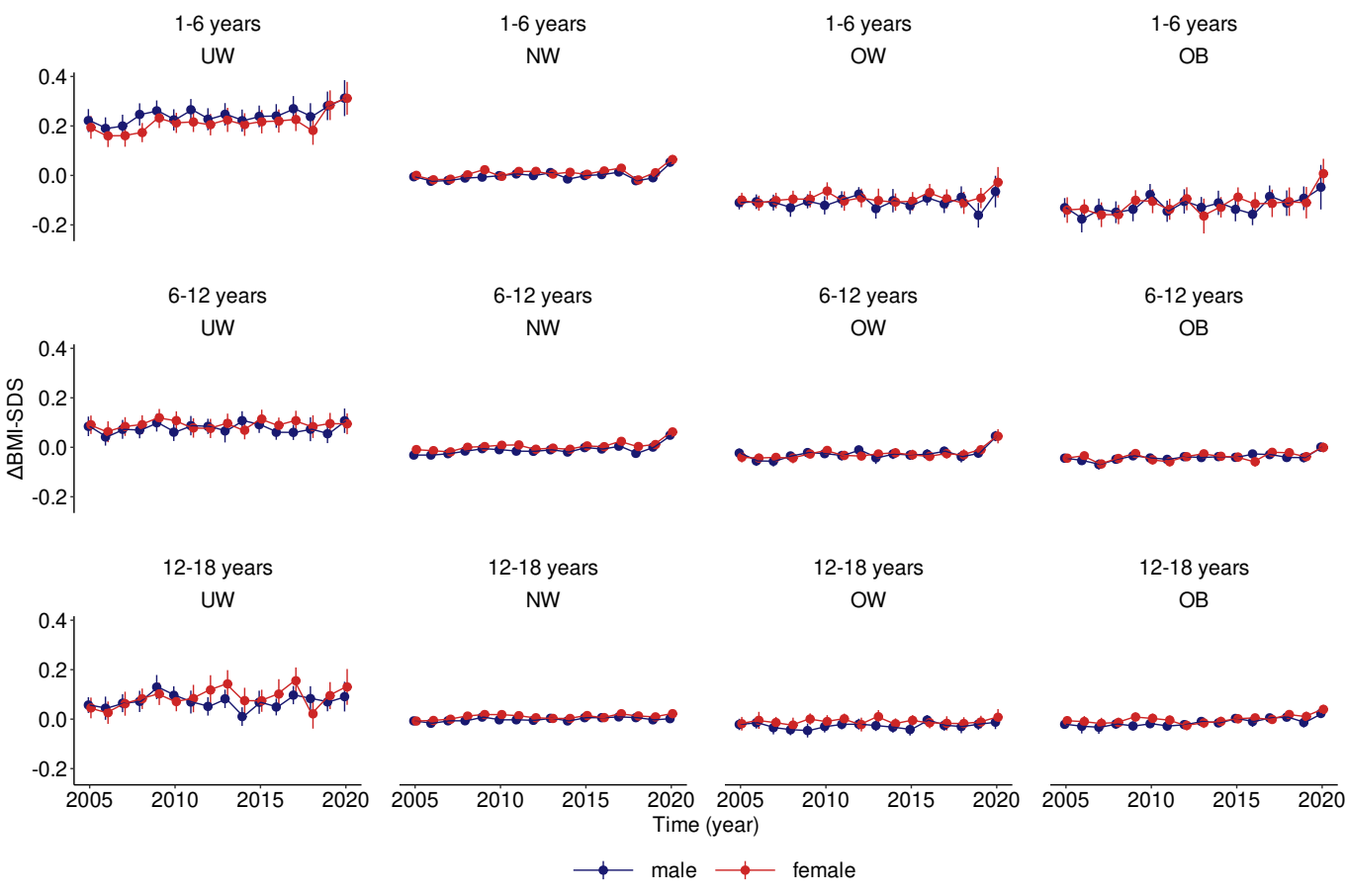


eFigure 2: Trends of the proportion of children gaining weight within the age and weight groups stratified by sex: Interestingly, we found no difference in trends between boys and girls.


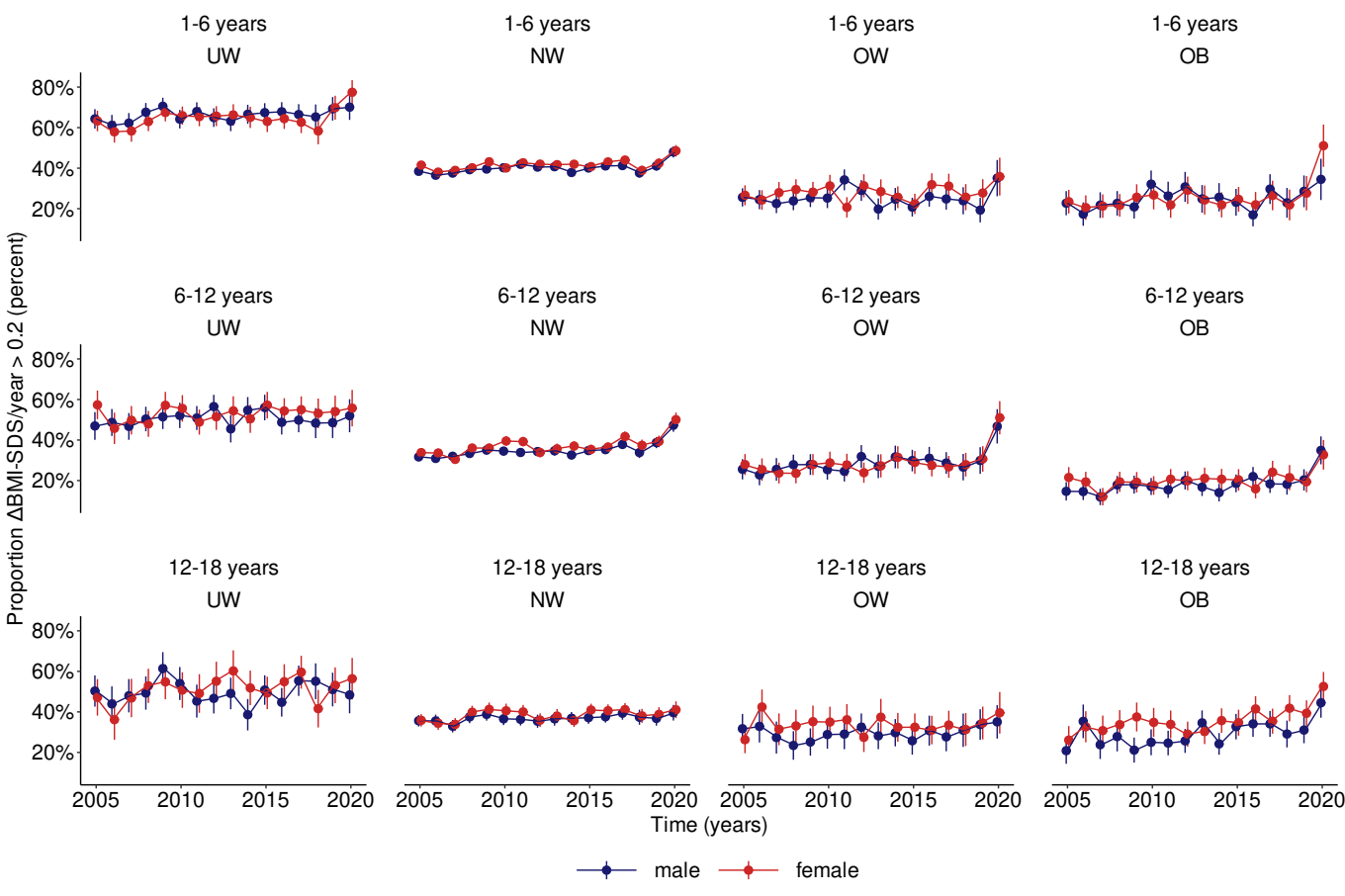


eFigure 3: Trends of the proportion of children losing weight within the age and weight groups stratified by sex: Interestingly, we found no difference in trends between boys and girls.


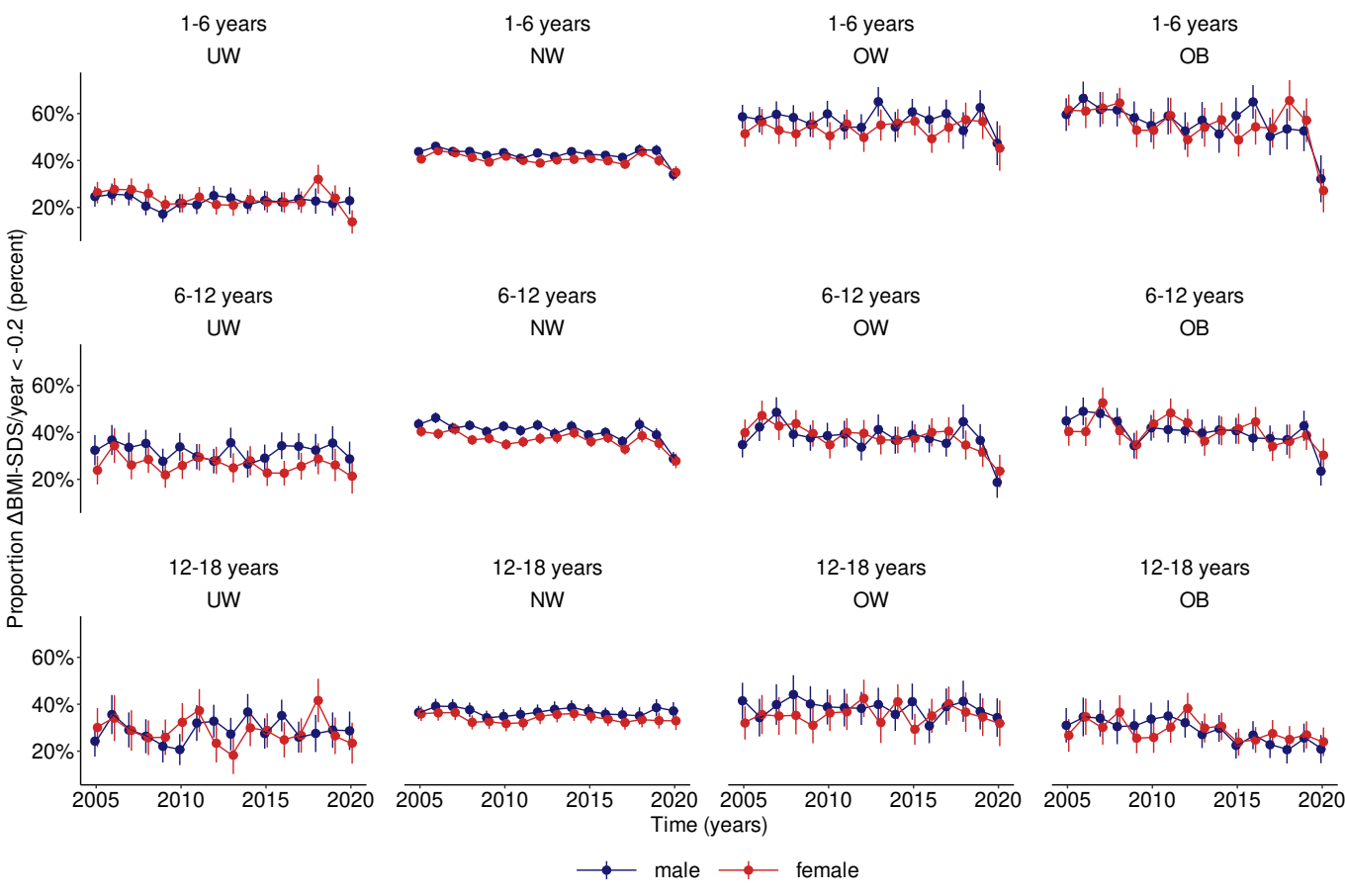

Supplement: Supplementary file 1 — Supplementary Material [file 41366_2021_968_MOESM1_ESM.docx]
